# Association between dietary diversity in complementary feeding and growth and nutritional status among children aged 0–36 months in China: a cross-sectional study

**DOI:** 10.3389/fpubh.2026.1775120

**Published:** 2026-09-24

**Authors:** Li Hong, Yunman Liu, Qihui Shao, Yizhi Wang, Chengchen Zhang, Liya Pan, Zhuoqi Zhao, Yuyun Chen

**Affiliations:** 1 Shanghai Children's Medical Center, Shanghai, China; 2 Fujian Children’s Hospital, Fuzhou, China

**Keywords:** children, complementary feeding, dietary diversity, growth and weight status, parental perception

## Abstract

**Objective:**

To assess complementary feeding practices among Chinese parents and to investigate the associations between dietary diversity in complementary feeding and growth outcomes in children aged 0–36 months.

**Methods:**

This national cross-sectional study included 26,266 children aged 0–36 months from 33 provinces in China. Participants were recruited through a convenience sampling strategy. Self-reported questionnaires were designed and posted on the website of the China Maternal and Child Health Association, which collected the information of assessing complementary feeding practices and knowledge. Anthropometric indicators (length/height, weight, and weight-for-length/height) were evaluated using World Health Organization growth standards. Dietary diversity was defined based on the daily consumption of multiple complementary food groups. Correlation analyses and multivariable regression models were conducted to evaluate associations between dietary diversity, specific food groups, and growth outcomes.

**Results:**

A high proportion of children were classified above the 90th percentile for weight across regions (range: 39.39–51.83%). Dietary diversity was positively associated with linear growth in all age groups, with the strongest association observed among children aged 19–36 months (*r* = 0.2769, *p* < 0.05). Conversely, dietary diversity was negatively associated with weight-for-length/height in children older than 6 months and with weight gain among those aged 13–18 months (*r* = −0.0602, *p* < 0.05). Iron and protein-rich foods, such as liver and animal blood, were underutilized, particularly in younger children. Additionally, 13.13% of parents reported limited experience in complementary feeding, and more than half prioritized DHA supplementation over whole-food diversity.

**Conclusion:**

Chinese children aged 0–36 months may be at risk of high body weight status. Greater dietary diversity in complementary feeding may support optimal linear growth while helping to mitigate excessive weight gain. Given the observed gap between parental knowledge and feeding practices, targeted nutrition education and guidance on responsive feeding are urgently needed from a public health perspective.

## Introduction

Complementary feeding refers to the process of gradually introducing solid foods in addition to breastfeeding or formula feeding for children aged 6–36 months, a key stage in which to establish a healthy diet (1). The first two years of life are very important in both the short and long term, as they represent the peak period for malnutrition and the risk of developing common childhood illnesses (2). The World Health Organization (WHO) updated its global guidelines in 2023, emphasizing timely introduction and dietary diversity. A key indicator of this is minimum dietary diversity (MDD), defined as consumption of at least five of eight food groups (3). Inappropriate complementary feeding (e.g., undesired eating patterns or large portion sizes) puts children at greater risk of developing overweight, obesity (4), and subsequent cardiometabolic diseases in their future lives (5).

Beyond weight status, the composition of complementary foods also affects neurodevelopment: protein, iron, zinc, polyunsaturated fatty acids (PUFAs), arachidonic acid (AA), and docosahexaenoic acid (DHA) are linked to better cognitive and motor outcomes (6–8). Despite growing parental concern about nutrition and weight management in China, the prevalence of overweight and obesity in children under 6 years has tripled over the past three decades (9). A recent national survey reported that among children aged 6–23 months in urban early education settings, the prevalence of MDD, minimum meal frequency, and minimum acceptable diet were only 59.4, 60.6, and 39.2%, respectively, while non-responsive feeding practices were common (28–64%) (10). These figures suggest that improper complementary feeding remains a serious challenge in China.

However, existing studies have largely focused on specific populations (e.g., urban infants) and the relationships between complementary feeding practices, child growth trajectories, and parental knowledge across a broader age range (0–36 months) and diverse settings in China have not been investigated in a large population. Moreover, the gap between parental knowledge and feeding practices in reality is underexplored. Therefore, the aim of our study was to investigate complementary feeding practices in children aged 0–36 months in China, the relationships between complementary feeding practices and growth of children, and parents’ knowledge in this field.

## Methods

### Study design

This population-based cross-sectional study was conducted using data from a national online survey on complementary feeding knowledge and practices in China. The self-reported questionnaire was developed by registered dietitians and clinicians and posted on the website of the China Maternal and Child Health Association where pediatricians from all the provinces have access to. Convenience sampling was used to recruit children and parents when they visited the pediatricians’ clinic between January 1 and January 10, 2022.

Eligible participants were children aged 0–36 months who were born full-term (37–42 weeks of gestation) with a normal birth weight (2,500–4,000 g). Children were excluded if they were older than 36 months, from multiple births, had congenital anomalies, genetic disorders, and severe illnesses at the time of survey, or incomplete questionnaire data. After data cleaning, 26,266 children were included in the final analysis.

This study was conducted under the ethical approval of Institutional Review Board of Shanghai Children’s Medical Center Affiliated to Shanghai Jiao Tong University School of Medicine (No. SCMCIRB-K2024019-1).

### Data collection

The questionnaire consisted of four sections: (1) demographic information (sex, date of birth, region, and family structure); (2) child growth, including parent-reported length/height and weight; (3) complementary feeding practices, including food groups introduced, snack consumption, and dietary supplement use; and (4) parental perceptions and knowledge regarding complementary feeding.

### Growth assessment

Anthropometric indicators—length/height, weight, and weight-for-length/height (WFL/H)—were assessed using WHO growth standards. Children were categorized into percentile groups (<10th, 10th–25th, 25th–50th, 50th–75th, 75th–90th, and ≥90th percentiles) for each indicator. “High weight status” is defined as having a body weight greater than the 75th percentile.

### Dietary diversity

To borrow the definition of MDD from Infant and Young Child Feeding Practices (2021), in our study, dietary diversity in complementary feeding was defined as the daily consumption of more than five food groups among the following categories: starches; liver and animal blood; fruits; vegetables; meat; seafood (fish and shrimp); beans; and eggs. This definition was adapted from WHO infant and young child feeding indicators and aligned with Chinese dietary guidelines.

### Statistical analysis

Descriptive statistics were used to summarize participant characteristics and feeding practices. Spearman correlation coefficients were calculated to assess associations between dietary diversity and growth indicators. Logistic regression models were applied to assess associations between individual food groups and achieving length/height, weight, or WFL/H within the 25th–75th percentile range, adjusting for age and sex. All analyses were performed using SAS version 9.4, with statistical significance set at *p* < 0.05.

## Results

### Basic characteristics of the subjects

A total of 32,217 children participated in the survey. Those with incomplete questionnaires, those older than 36 months, or those whose height or weight was incorrectly reported, defined as the length or weight of children smaller than the 0.1th percentile of 0 months of age or larger than the 99.9th percentile of 36 months of age, were excluded. Finally, 26,266 children were enrolled (Figure 1) and further divided into the following four groups according to their age: the 0 to 6-month age group, 7 to 12-month age group, 13 to 18-month age group and 19 to 36-month age group (Table 1). The 7 to 12-month age group had the largest population (*n* = 18,026, 68.63%) among the age groups. Most children in this study lived in East China (*n* = 9,648, 36.73%), the economically developed region in China, followed by South China (*n* = 4,686, 17.82%) and Central China (*n* = 4,512, 17.18%). Owing to the limited sample size, data from Hong Kong, Macao, and Taiwan were not considered in the following analysis. The sex ratio of children was 1 to 1. Most of the families participating in this study had only one child.

**Figure 1:**
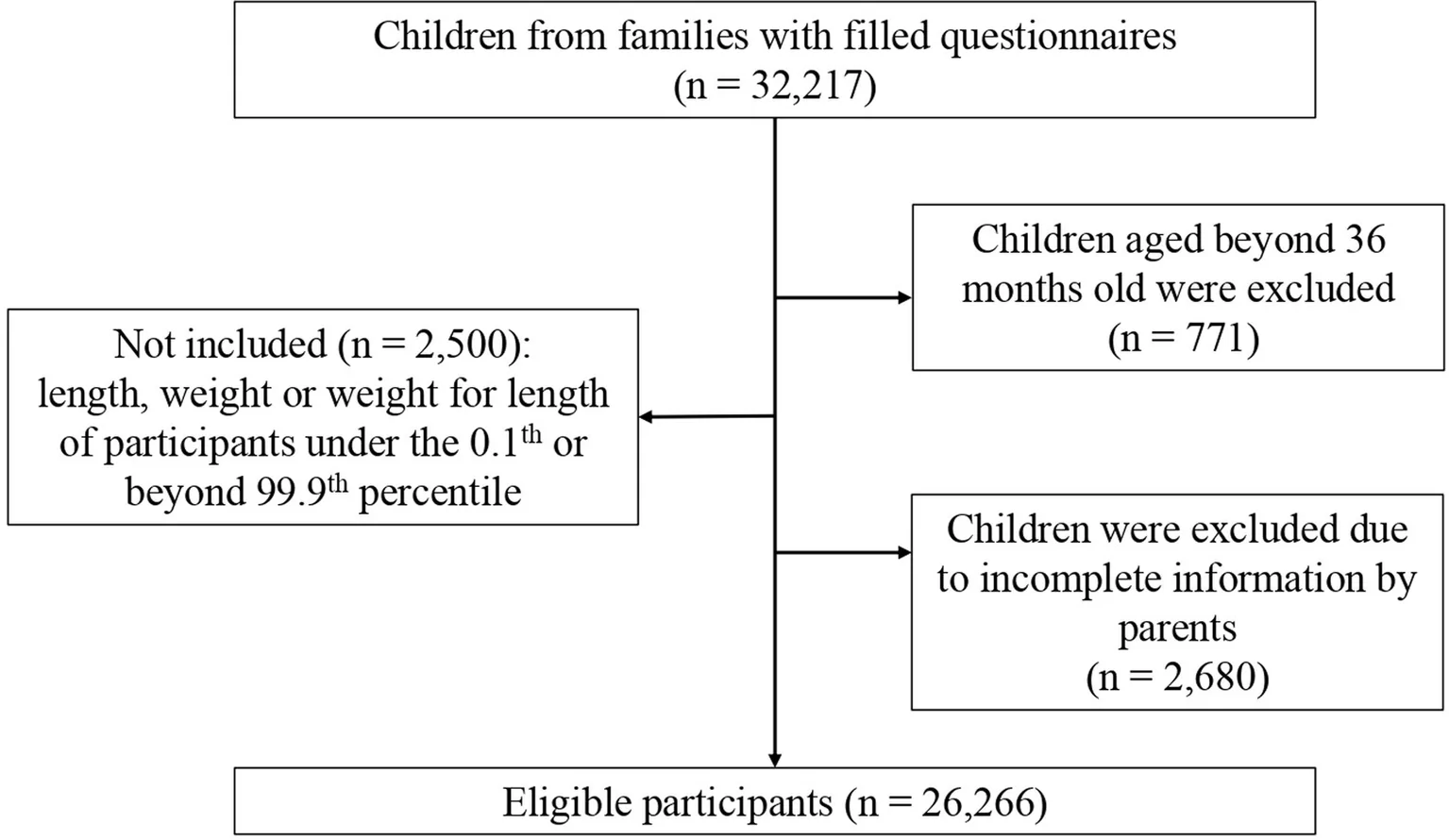
Study flowchart.

**Table 1:** Basic characteristics of the participants.

	Total	Months of Age (*N*, %)			
		0–6	7–12	13–18	19–36
N	26,266 (100.00)	4,524 (17.22)	18,026 (68.63)	2,376 (9.05)	1,340 (5.10)
Gender					
Male	13,146 (50.05)	2,226 (49.20)	8,964 (49.73)	1,123 (47.26)	833 (62.16)
Female	13,120 (49.95)	2,298 (50.80)	9,062 (50.27)	1,253 (52.74)	507 (37.84)
Region					
Northeast China	1,476 (5.62)	343 (23.24)	932 (63.14)	113 (7.66)	88 (5.96)
North China	3,277 (12.48)	532 (16.23)	2,251 (68.69)	310 (9.46)	184 (5.61)
East China	9,648 (36.73)	1,513 (15.68)	6,733 (69.79)	888 (9.20)	514 (5.33)
Central China	4,512 (17.18)	757 (16.78)	3,124 (69.24)	422 (9.35)	209 (4.63)
South China	4,686 (17.84)	879 (18.76)	3,238 (69.10)	381 (8.13)	188 (4.01)
Southwest China	1,688 (6.43)	289 (17.12)	1,128 (66.82)	162 (9.60)	109 (6.46)
Northwest China	974 (3.71)	211 (21.66)	617 (63.35)	99 (10.16)	47 (4.83)
Hong Kong, Macao and Taiwan	5 (0.02)	0 (0.00)	3 (60.00)	1 (20.00)	1 (20.00)
Number of children in the family					
1	18,899 (71.95)	3,148 (69.58)	13,445 (74.59)	1,549 (65.19)	757 (56.49)
2	6,076 (23.13)	995 (21.99)	3,828 (21.24)	751 (31.61)	502 (37.46)
3 or more	1,291 (4.92)	381 (8.42)	753 (4.18)	76 (3.20)	81 (6.04)

In most regions, the proportion of children with length/height <p25 exceeded 22%; however, in the Northwest China, there were only 17.56% children with length/height <p25 (Table 2). In the length/height ≥p90 group, children in Northeast China represented the largest proportion (42.48%), whereas those in southern China constituted the smallest proportion (30.94%). In the weight ≥p90 group, the proportion ranged from 39.39% in South China to 51.83% in Northeast China. With respect to WFL/H, as an indicator of children’s weight health in relation to height, South China had the highest proportion of children with WFL/H < p25 (19.48%), whereas North China had the largest sample of children with WFL/H ≥ p90 (50.79%). At least 30% of the children in each region had WFL/H ≥ p90, especially in North China (51.83%).

**Table 2:** Distribution of growth percentile in children aged 0–36 months old in China.

	Region	Growth and development status (*N*, %)					
		<p10	p10-p25	p25-p50	p50-p75	p75-p90	≥p90
Length	Northeast China	263 (17.82)	86 (5.83)	112 (7.59)	211 (14.30)	180 (12.20)	627 (42.48)
	North China	553 (16.88)	240 (7.32)	314 (9.58)	474 (14.46)	441 (13.46)	1,255 (38.30)
	East China	1,547 (16.03)	576 (5.97)	917 (9.50)	1,582 (16.4)	1,389 (14.40)	3,637 (37.70)
	Central China	750 (16.62)	281 (6.23)	451 (10.00)	770 (17.07)	575 (12.74)	1,685 (37.34)
	South China	734 (15.66)	395 (8.43)	590 (12.59)	813 (17.35)	704 (15.02)	1,450 (30.94)
	Southwest China	274 (16.23)	122 (7.23)	177 (10.49)	307 (18.19)	227 (13.45)	581 (34.42)
	Northwest China	120 (12.32)	51 (5.24)	95 (9.75)	170 (17.45)	140 (14.37)	398 (40.86)
Weight	Northeast China	53 (3.59)	47 (3.18)	141 (9.55)	248 (16.80)	222 (15.04)	765 (51.83)
	North China	94 (2.87)	106 (3.23)	354 (10.80)	586 (17.88)	518 (15.81)	1,619 (49.4)
	East China	326 (3.38)	263 (2.73)	1,050 (10.88)	1,858 (19.26)	1,397 (14.48)	4,754 (49.27)
	Central China	164 (3.63)	170 (3.77)	528 (11.70)	832 (18.44)	629 (13.94)	2,189 (48.52)
	South China	254 (5.42)	288 (6.15)	779 (16.62)	947 (20.21)	572 (12.21)	1,846 (39.39)
	Southwest China	72 (4.27)	81 (4.80)	236 (13.98)	338 (20.02)	238 (14.10)	723 (42.83)
	Northwest China	31 (3.18)	34 (3.49)	116 (11.91)	191 (19.61)	150 (15.40)	452 (46.41)
Weight for length	Northeast China	135 (9.16)	72 (4.88)	163 (11.06)	187 (12.69)	182 (12.35)	736 (49.86)
	North China	266 (8.12)	187 (5.71)	386 (11.79)	424 (12.95)	348 (10.63)	1,664 (50.79)
	East China	741 (7.68)	463 (4.80)	1,222 (12.67)	1,381 (14.31)	1,087 (11.27)	4,755 (49.28)
	Central China	379 (8.41)	284 (6.30)	547 (12.13)	642 (14.22)	485 (10.76)	2,174 (48.19)
	South China	530 (11.3)	383 (8.18)	715 (15.25)	744 (15.87)	444 (9.48)	1,871 (39.92)
	Southwest China	174 (10.31)	110 (6.52)	223 (13.22)	266 (15.77)	188 (11.14)	726 (43.03)
	Northwest China	85 (8.73)	67 (6.88)	127 (13.04)	146 (14.99)	114 (11.70)	435 (44.66)

<p10, < the 10th percentile; p10-p25, ≥ the 10th but < 25th percentile; p25-p50, ≥ the 25th but < 50th percentile; p50-p75, ≥ the 50th but < 75th percentile; p75-p90, ≥ the 75th but < 90th percentile; ≥ p90, ≥ the 90th percentile.

### Complementary feeding

The dietary patterns of each age group were assessed in this study (Figure 2). Cereal-based products clearly dominated the complementary foods for children in China (in the 0 to 6-month age group, *n* = 3,786, 83.7%; in the 7 to 12-month age group, *n* = 15,322, 85.0%; in the 13 to 18-month age group, *n* = 2,117, 89.1%; in the 19 to 36-month age group, *n* = 1, 149,85.8%), followed by fruits and vegetables. The proportion of children fed with liver or animal blood was not satisfactory. Fifty-five percent of the children in the 19 to 36-month age group were given liver and animal blood, far fewer than the number of children given food from other food groups.

**Figure 2:**
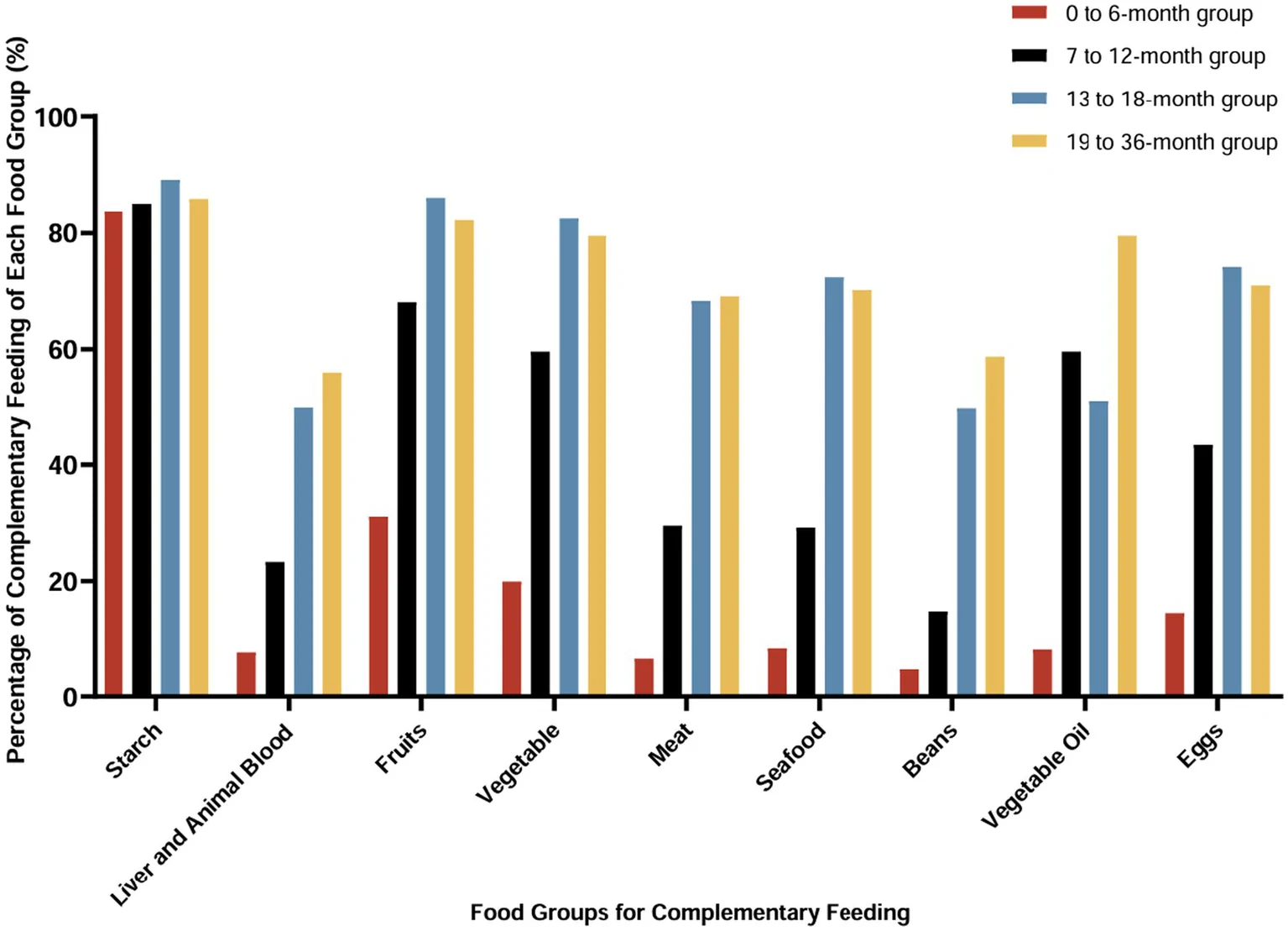
Food groups for complementary feeding in children aged 0–36 months.

The introduction of snacks and dietary supplements to children of different ages was further investigated (Supplementary Figures S1, S2). Wheat products were mostly consumed (in the 13 to 18-month age group, *n* = 1,708, 71.9%; in the 19-to 36-month age group, *n* = 902, 67.3%), followed by rice products (in the 13 to 18-month age group, *n* = 1,663, 70.0%; in the 19 to 36-month age group, *n* = 835, 62.3%). The consumption of dairy snacks and frozen fruits and vegetables was lower overall than that of cereal-based products. Among the children aged 13–36 months, the 19 to 36-month age group had the lowest percentage of those consuming frozen fruits and vegetables (*n* = 682, 50.9%), whereas dairy products were the least prevalent in the 13 to 18-month age group (*n* = 969, 40.8%). Dietary supplements were commonly used by all the children in this study, with vitamin AD complexes and vitamin D being taken most frequently. A higher rate of vitamin AD supplementation was observed in younger children (in the 0 to 6-month age group, *n* = 2,909, 64.3%; in the 7 to 12-month age group, *n* = 10,581, 58.7%; in the 13 to 18-month age group, *n* = 1,314, 55.3%; in the 19 to 36-month age group, *n* = 656, 49.0%).

### Association between growth and complementary feeding

We further analysed the correlation between intake of complementary foods and growth of children among each age group.

In terms of length or height (Supplementary Figure S3), all the food groups except beans were likely to benefit infants at the p25-p75 for length in the 7 to 12-month age group. After adjustment for age, sex and food groups, significant associations between achieving the p25-p75 for height in the 13 to 18-month age group and vegetable (OR, 1.35; 95% CI, 1.01 to 1.82) and seafood (e.g., fish and shrimp) (OR, 1.39; 95% CI, 1.07 to 1.81) consumption were detected. With the increase of age, there was no significant association between complementary feeding with starch and height reaching height p25-p75.

Moreover, feeding infants with fruit was associated with reaching weight p25-p75, except in the 0 to 6-month age group (Supplementary Figure S4). After adjustment for age, sex and food groups, weight in the p25-p75 might be associated with complementary feeding with fruit (OR, 1.54; 95% CI, 1.02 to 2.32) and seafood (OR, 1.54; 95% CI, 1.07 to 2.22) in the 13 to 18-month age group and with liver and animal blood (OR, 1.44; 95% CI, 1.06 to 1.97), beans (OR, 1.81; 95% CI, 1.23 to 2.68), and eggs (OR, 1.52; 95% CI, 1.05 to 2.21) in the 18 to 36-month age group.

With respect to WFL (Supplementary Figure S5), all the food groups except beans were associated with WFL in the p25-p75 in the 7 to 12-month age group. After adjustment for age, sex and food groups, surprisingly, protein-rich foods, e.g., liver and animal blood, seafood and eggs, were not significantly associated with WFL p25-p75. Instead, the intake of cereal (OR, 1.73; 95% CI, 1.55 to 1.92) or fruits (OR, 1.22; 95% CI, 1.11 to 1.33) was more strongly associated with attaining WFL p25-p75 in the 7 to 12-month age group.

In addition, the associations between dietary diversity and growth were analysed (Table 3). Dietary diversity was positively associated with length/height in all age groups, especially in the 19 to 36-month age group (*r* = 0.2769, *p* < 0.05). Dietary diversity was negatively associated with weight in the 13 to 18-month age group (*r* = −0.0602, *p* < 0.05) and WFL, but not in the 0 to 6-month age group. Therefore, while the association between any single food group and growth demonstrated no particularly significant advantage, improving dietary diversity through the inclusion of appropriate food groups could effectively promote children’s growth. Additionally, this approach offered potential benefits in preventing children from reaching a high weight status.

**Table 3:** The correlation coefficient between dietary diversity and length, weight, and weight for length.

	Months of Age			
	0–6	7–12	13–18	19–36
Length	0.0416*	0.1140*	0.1012*	0.2769*
Weight	0.0478*	−0.0097	−0.0602*	0.0887*
Weight for length	−0.0113	−0.0939*	−0.1069*	−0.1603*

*p-value < 0.05.

### Perception of complementary feeding in parents

We further evaluated parental knowledge of complementary feeding (Table 4). Parents in all age groups agreed that the reasons for introducing complementary foods were to perform natural chewing movements, followed by preparing for a gradual transition from liquid food to conventional solid food. In addition, the most important factors related to the choices of complementary foods by parents were months of age and developmental stage, including digestive function (*n* = 18,181, 69.22%). Compared with parents in the other three groups, parents in the 19 to 36-month age group (*n* = 295, 22%) were more concerned about high nutrient-density foods, e.g., foods rich in calcium, iron, or zinc. DHA was given priority by more than half of the parents, especially those of children in the 0 to 6-month age group (*n* = 3,248, 71.80%).

**Table 4:** Perception of complementary feeding in parents.

	Months of age (*N*, %)			
	0–6	7–12	13–18	19–36
Reasons to add complementary foods				
Limited nutrients in breast milk	2,488 (55.00)	9,734 (54.00)	1,259 (52.99)	630 (47.01)
Exercise in chewing and swallowing food	3,393 (75.00)	14,601 (81.00)	1,901 (80.01)	1,005 (75.00)
Stimulate taste development	2,488 (55.00)	10,635 (59.00)	1,473 (61.99)	777 (57.99)
Stimulate olfactory development	1,900 (42.00)	8,292 (46.00)	1,236 (52.02)	657 (49.03)
Stimulate visual development	1,493 (33.00)	6,850 (38.00)	1,069 (44.99)	590 (44.03)
Exercise hand-eye coordination and fine motor skills	1,991 (44.01)	9,374 (52.00)	1,568 (65.99)	831 (62.01)
Preparing for the adult diet	2,850 (63.00)	11,717 (65.00)	1,639 (68.98)	898 (67.01)
Top factors in selecting complementary foods				
Speculation about children’s taste preferences	516 (11.40)	2,109 (11.70)	321 (13.50)	218 (16.30)
Age and digestive capacity	3,135 (69.30)	12,636 (70.10)	1,601 (67.40)	809 (60.40)
High nutrient content	805 (17.80)	3,137 (17.40)	432 (18.20)	295 (22.00)
Others	68 (1.50)	144 (0.80)	21 (0.90)	17 (1.30)
Nutrients of concern				
Good quality fat	1,054 (23.30)	4,362 (24.20)	623 (26.20)	373 (27.80)
Lutein	1,493 (33.00)	5,642 (31.30)	748 (31.50)	482 (36.00)
Dietary fiber	2,090 (46.20)	8,562 (47.50)	1,240 (52.20)	704 (52.50)
Docosahexaenoic acid	3,248 (71.80)	12,330 (68.40)	1,649 (69.40)	930 (69.40)
Arachidonic acid	774 (17.10)	2,974 (16.50)	392 (16.50)	268 (20.00)
Lactoferrin	1,335 (29.50)	4,921 (27.30)	649 (27.30)	410 (30.60)
Anthocyanin	579 (12.80)	2,073 (11.50)	321 (13.50)	200 (14.90)
Parents’ awareness of exercising children’s chewing ability in complementary feeding				
Consciousness	3,131 (69.20)	14,295 (79.30)	1,963 (82.60)	1,127 (84.10)
Unconsciousness	502 (11.10)	1,532 (8.50)	178 (7.50)	90 (6.70)
Unaware of how to cultivate	891 (19.70)	2,199 (12.20)	235 (9.90)	123 (9.20)
Parents’ perceptions of the significance of practicing chewing				
Favours the development of gastrointestinal function	3,809 (84.20)	14,799 (82.10)	1,993(83.90)	1,096 (81.80)
Helps eruption of deciduous teeth	2,493 (55.10)	10,545 (58.50)	1,447(60.90)	728 (54.30)
Helps with sound and language development	2,665 (58.90)	11,140 (61.80)	1,618(68.10)	886 (66.10)
Self-directed eating builds self-confidence	3,027 (66.90)	11,879 (65.90)	1,763(74.20)	1,018 (76.00)

In practice, some parents lacked the skills to cultivate their children’s chewing ability through complementary feeding (*n* = 3,448, 13.13%). Inexperienced parents were found in the younger age group (0 to 6-month age group, *n* = 891, 19.7%). Moreover, parents of older children paid more attention to the ability to feed themselves and self-confidence through complementary feeding (19 to 36-month age group, *n* = 1,018, 76.0%). However, among parents with children in the 19 to 36-month age group, only 218 (16.3%) parents considered their children’s different tastes and preferences. Therefore, parents generally paid attention to the transitional function and nutritional requirements during complementary feeding, but less attention to the cultivation of children’s chewing ability and personalized dietary preferences. In addition, as children grew older, parents’ concerns about high-nutrient density food and self-care ability training had increased significantly, but there were still insufficient experience and skills in the process of introducing complementary foods.

## Discussion

This was a population-based cross-sectional study in China that included children aged 0–36 months. This study aimed at investigating the current complementary feeding practices in China and their associations with children’s growth and parental perceptions and knowledge. Elevated risk of overweight and obesity among Chinese children possibly due to inappropriate complementary feeding was identified, while dietary diversity demonstrated dual benefits in supporting growth and preventing high weight status in children aged 0–36 months. In the process of adding complementary foods, the gap remained between parents’ awareness and action, which required further guidance from clinicians or nutritionists.

Over the past few decades, the overall prevalence of malnutrition among preschool children in China has decreased significantly, but it remains high among those at the age when complementary foods are introduced (11). A national representative survey in China suggested that the dietary diversity and meal frequency of children aged 6–23 months were not optimal during complementary feeding, and that an increasing trend of stunting and anaemia rates was observed (12). Studies have indicated inadequate intake of protein-rich foods and dairy-based patterns when children aged 12 to 23 months in China were introduced complementary foods (13). Another national survey showed that the energy and nutrient intakes from complementary foods among children aged 12 to 23 months in central China were low (14). Therefore, the unreasonable addition of complementary foods to children in China exists, and it is urgent to understand the current situation of complementary feeding among children in China through large-scale research, and to provide guidance for the public.

In this study, we first assessed the current practice of complementary feeding in Chinese children aged 0–36 months. Approximately 17% of parents started complementary feeding for their children younger than 6 months of age. Research findings from the United States (15) and Ireland (16) indicate that approximately 20% of mothers introduced complementary foods before 4 months of age. In Europe, the timing of introduction of complementary foods was before 6 months of age (17), while postponed in India (18). Disparities across culture and geographies may be related to multiple factors, e.g., age and educatio of the mothers, the socioeconomic status of the caregivers, the influence of social media, postpartum care, and lack of professional consultation (19). Cereal, as the first food for complementary feeding, seemed to be common among parents worldwide. In this study, more than 80% of the parents chose cereal-based products as the first complementary food for their children. 74% of parents in the UK (20) and 63.9% in Italy (21) preferred ground rice or wheat. This might be because of the culture and parental concerns of the immature digestive system and the weak chewing ability of infants (22). In addition, in this study, the percentage of introduction of animal blood or liver was lower than that of the other food groups regardless of age, especially among children younger than 12 months of age. This is similar to the results of previous national surveys. However, the Dietary Guidelines for Chinese (2022) recommended that iron-rich foods, e.g., meat purée, liver purée, and iron-fortified cereal, be introduced first for complementary feeding. Liver and animal blood are rich in iron and protein, preventing anaemia and supporting immune and neurological functions; moreover, liver is an important food source of vitamin A, which helps maintain normal vision, support the immune system and growth and development. Thus, education on complementary feeding for parents in China should reinforce the concept that iron-rich foods be introduced first.

Moreover, we investigated the growth of Chinese children aged 0–36 months, and further explored its correlation with complementary feeding. A remarkable proportion of children with large weights were observed in this study, which calls for urgent attention, as childhood obesity has been a critical issue globally (23). Children above the 90th percentile for weight were mainly from economically developed regions, while the northwest region had the largest sample of undernourished children in our study. Fruits appeared to be a favoured complementary food by parents, but their intake was associated with weight gain instead of length/height growth in children. Fish and shrimp, additionally, promoted the weight growth of children aged 7–18 months. A similar result was obtained by Chipili et al., who reported that fish consumption promoted linear weight growth in infants (24). Also, in our study, egg feeding promoted length/height, weight and WFL/H in the p25-p75 group. Similar findings from a randomized trial revealed that egg feeding (one egg per day for 6 months) could reduce the risk of stunting and the intake of sweetened foods (24). Furthermore, we introduced the concept of dietary diversity. In our study, a diverse complementary diet had a significantly favorable effect on length/height growth irrespective of age; this benefit was more pronounced at older ages. Meanwhile, dietary diversity was positively associated with weight growth in all the groups except the 7 to 12-month age group but negatively associated with WFL/H, except in the 0 to 6-month age group. These findings suggested that improving the diversity of complementary foods might prevent children from being at high weight status. According to the WHO guidelines, MDD‌ measures whether children aged 6–23 months have eaten foods from at least five distinct categories within the preceding day, based on eight predefined groups: breast milk; grains, roots, tubers and plantains; pulses (beans, peas, lentils), nuts and seeds;dairy products (milk, infant formula, yogurt, cheese); flesh foods (meat, fish, poultry, organ meats); eggs; vitamin-A rich fruits and vegetables; and other fruits and vegetables (25). According to the recommendations of the Dietary Guidelines for Chinese (2022), consuming at least four of the seven food groups every day guarantees MDD, i.e., grains, roots, tubers and plantains; pulses, nuts and seeds; dairy products; flesh foods; eggs; vitamin A-rich vegetables and fruits; and other vegetables and fruits (25–27). Research from China (28) and Iran (29) has shown that poor dietary diversity increased the risk of childhood obesity. The American Heart Association emphasized that the goal of dietary diversity should be achieved by selective foods, i.e., foods recommended by the WHO rather than sugary snacks (30). Therefore, proper management of fruit intake and improving dietary diversity within recommended food groups by the WHO may contribute to prevention of high weight status including obesity.

Finally, we investigated the parents’ understanding of complementary feeding and found that there was a knowledge-action gap in parents with respect to complementary feeding practices. Most parents with children in all the age groups believed that complementary feeding was meant to develop chewing and swallowing skills in children. Nevertheless, parents in the younger age groups reported that training in chewing and swallowing performance during complementary feeding was more challenging. In addition, 19.7% parents were unaware of how to cultivate children’s chewing ability in complementary feeding. Therefore, parental education on complementary feeding skills in early childhood is strongly needed for better preparation for taking care of young children. In addition, Black et al. reported that children allowed by their parents to decide on the timing, quantity, and rhythm of meals were more likely to develop a sense of independence and secure attachment (31). In this study, however, many parents overlooked the food preferences of their children when they chose complementary foods. Even among the parents of children aged 19–36 months, only 16.3% took their children’s tastes and preferences into account. Therefore, responsive feeding should be further promoted among parents in China, i.e., creating a good eating environment, preparing nutrient-dense foods for children on the basis of their age, and letting children decide what and how much to eat, followed by eating on their own (32).

Strengths of this study include its large, nationally diverse sample and comprehensive assessment of feeding practices and growth outcomes. Limitations include the cross-sectional design, reliance on parent-reported data, and the inability to account for socioeconomic factors such as parental education and household income.

In conclusion, Chinese children aged 0–36 months may be at risk of being at high weight status, ranging from high normal weight to obesity during early life. Adequate dietary diversity in complementary feeding may promote optimal linear growth while helping prevent excessive weight gain. Future public health strategies are sorely needed to be shifted toward actionable interventions, e.g., integrating dietary diversity consultation services into regular community medical services, promptly introducing iron-rich animal-based foods, and encouraging responsive feeding. Implementation and desirable outcomes would be guaranteed by trained health practitioners, accessible health education services and long-term monitoring of growth.

## Data Availability

The original contributions presented in the study are included in the article/Supplementary material, further inquiries can be directed to the corresponding author.
