# Supplementary material for: Association between dietary diversity in complementary feeding and growth and nutritional status among children aged 0–36 months in China: a cross-sectional study
Source: Front Public Health. 2026 Sep 24;14:1775120. doi: 10.3389/fpubh.2026.1775120 (PMC13647990; doi:10.3389/fpubh.2026.1775120)
Supplement: Supplementary file 1 [file Supplementary_file_1.pdf]

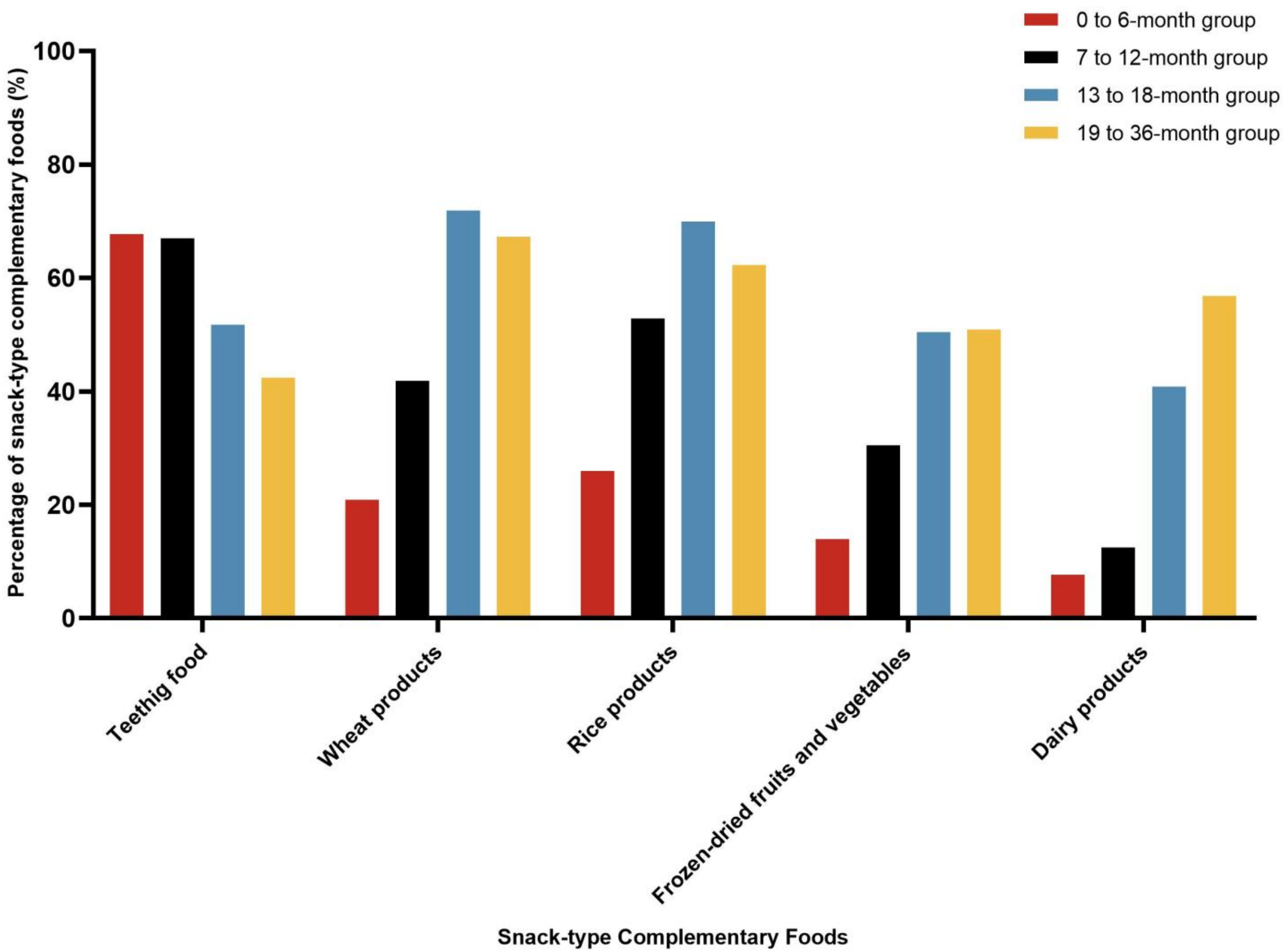

Supplementary Figure 1 Snacks for complementary feeding in children aged 0-36 months

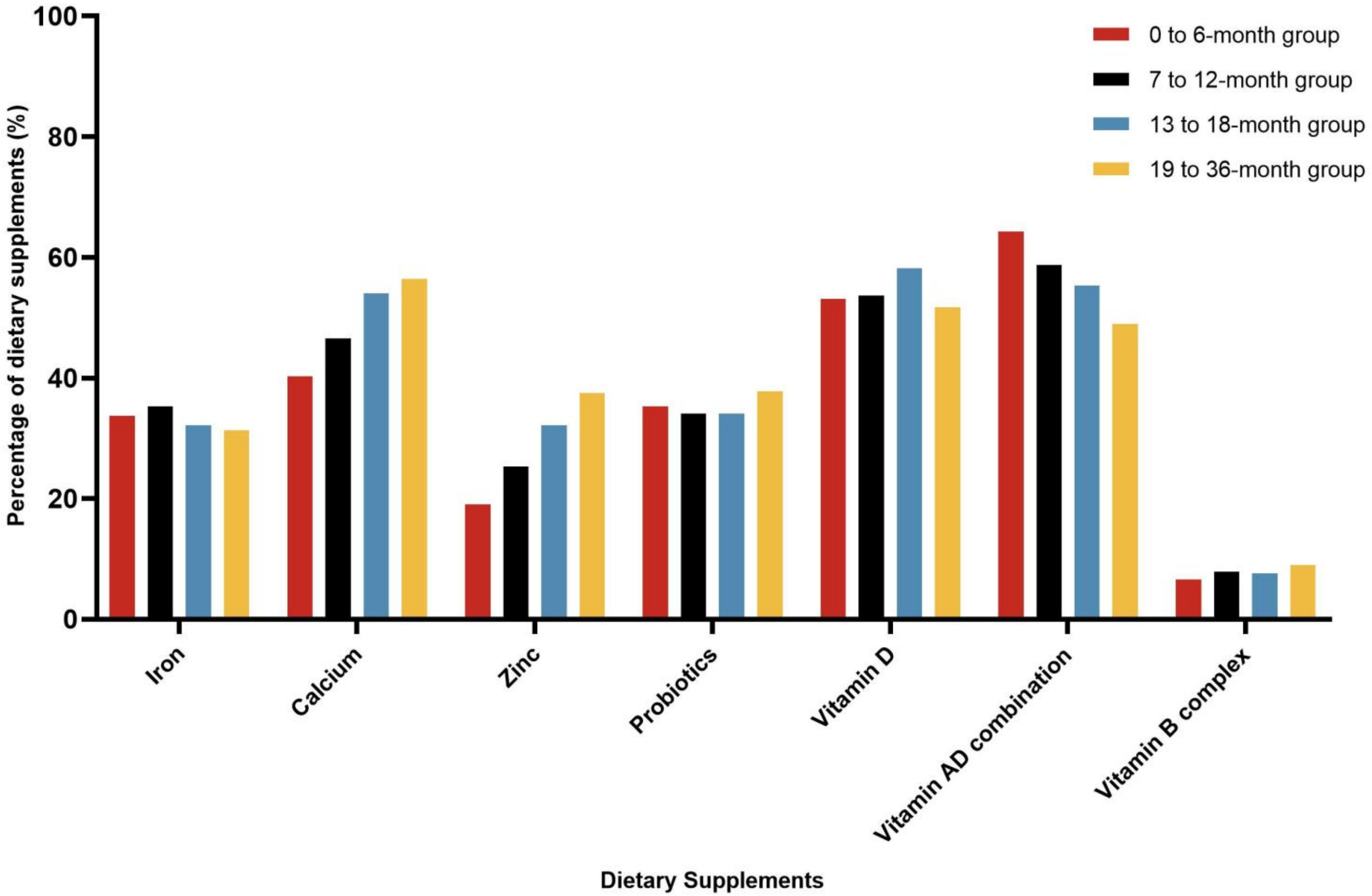

Supplementary Figure 2 Nutritional supplementation in children aged 0-36 months

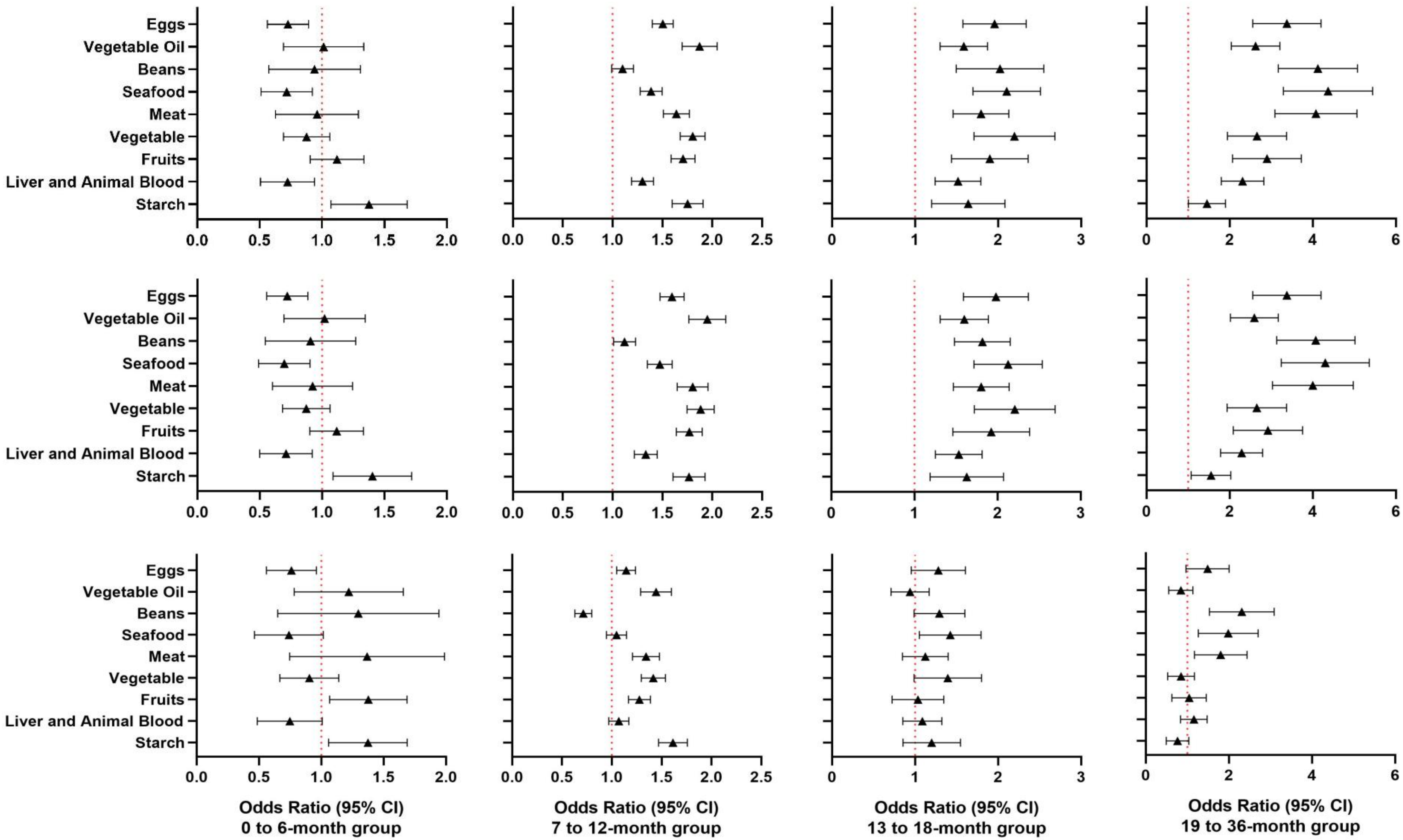

Supplementary Figure 3 Association between food groups and length in children aged 0-36 months old

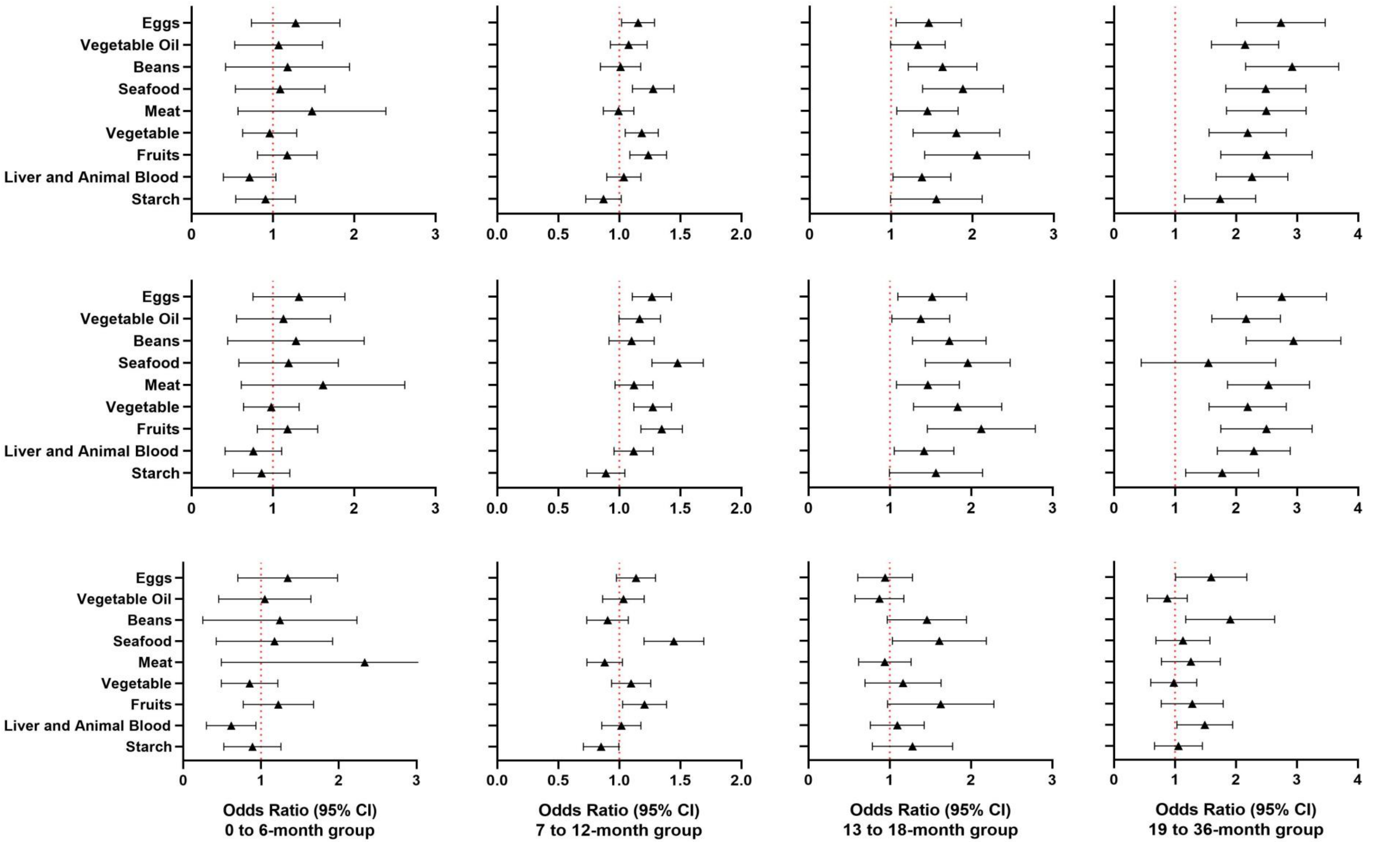

Supplementary Figure 4 Association between food groups and weight in children aged 0-36 months old

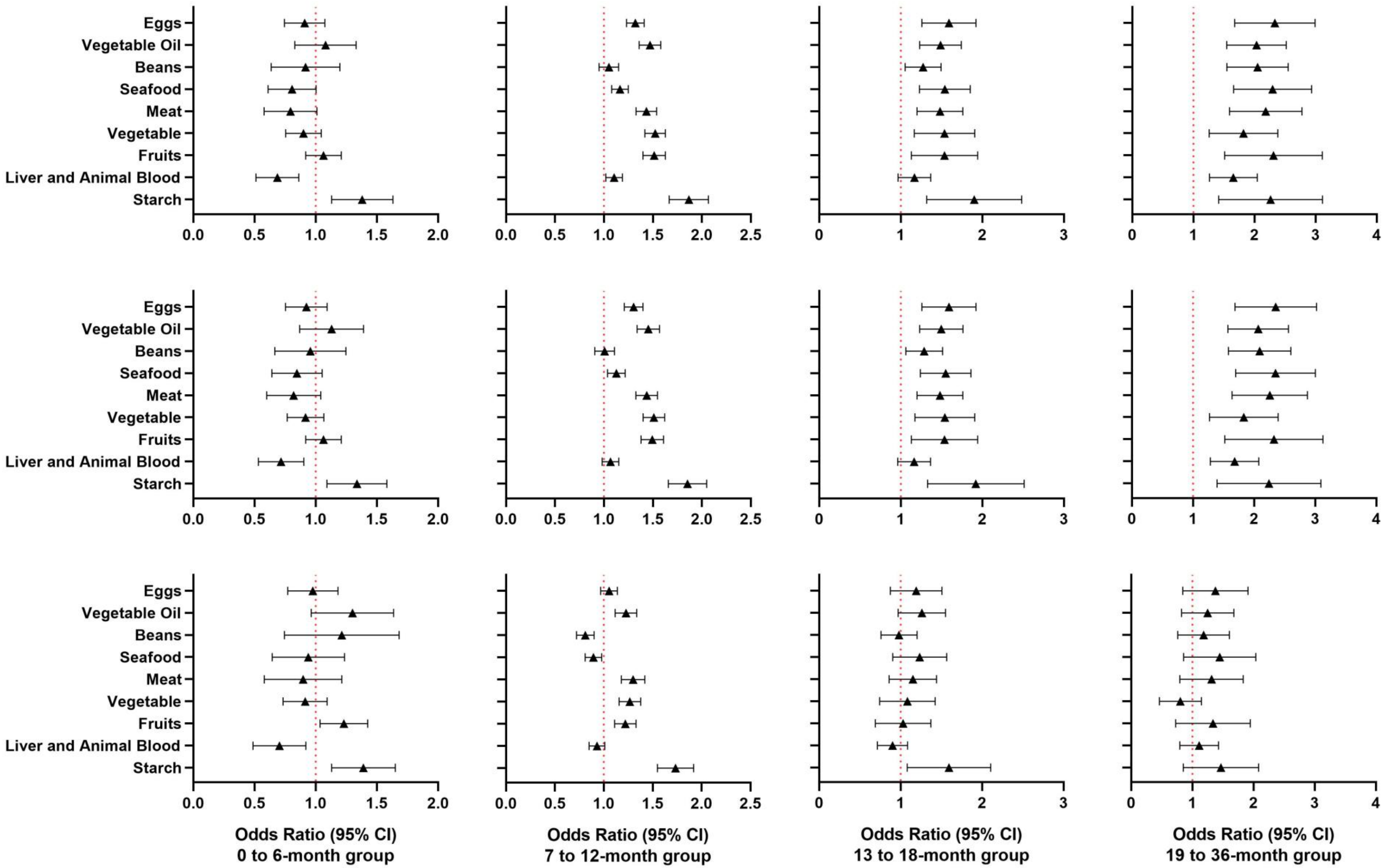

Supplementary Figure 5 Association between food groups and weight for length in children aged 0-36 months old
